# Modeling the Neurovascular Niche: Unbiased Transcriptome Analysis of the Murine Subventricular Zone in Response to Hypoxic Insult

**DOI:** 10.1371/journal.pone.0076265

**Published:** 2013-10-11

**Authors:** Qi Li, Sandra Canosa, Kelly Flynn, Michael Michaud, Michael Krauthammer, Joseph A. Madri

**Affiliations:** Department of Pathology, Yale University School of Medicine, New Haven, Connecticut, United States of America; Université Pierre et Marie Curie-Paris6, INSERM, CNRS, France

## Abstract

Premature infants often experience chronic hypoxia, resulting in cognitive & motor neurodevelopmental handicaps. These sometimes devastating handicaps are thought to be caused by compromised neural precursor cell (NPC) repair/recovery resulting in variable central nervous system (CNS) repair/recovery. We have identified differential responses of two mouse strains (C57BL/6 & CD1) to chronic hypoxia that span the range of responsiveness noted in the premature human population. We previously correlated several CNS tissue and cellular behaviors with the different behavioral parameters manifested by these two strains. In this report, we use unbiased array technology to interrogate the transcriptome of the subventricular zone (SVZ) in these strains. Our results illustrate differences in mRNA expression in the SVZ of both C57BL/6 and CD1 mice following hypoxia as well as differences between C57BL/6 and CD1 SVZ under both normoxic and hypoxic conditions. Differences in expression were found in gene sets associated with Sox10-mediated neural functions that explain, in part, the differential cognitive and motor responsiveness to hypoxic insult. This may shed additional light on our understanding of the variable responses noted in the human premature infant population and facilitate early intervention approaches. Further interrogation of the differentially expressed gene sets will provide a more complete understanding of the differential responses to, and recovery from, hypoxic insult allowing for more informed modeling of the ranges of disease severity observed in the very premature human population.

## Introduction

Despite continued advancements in perinatal care, preterm birth results in significant cognitive and motor disabilities. However, recent evidence suggests that there can be some recovery over time. Studies indicate that approximately 2% of the offspring of live births in the United States have birth weights under 1000 grams and their survival rates range from 60 to 85%. While survival rates of these infants have improved over time, neonatal illness and severe intraventricular hemorrhage (IVH) have increased, resulting in an increase of severely compromised patients that require care in neonatal intensive care units. Additionally, many very low birth weight preterm infants suffer from apnea and respiratory distress syndromes that result in cerebral hypoxemia. Of this group, one quarter have been found to function in the intellectually disabled or borderline ranges at school; 10% suffer from cerebral palsy; and at eight years of age one half require special assistance in school, resulting in annual life time care costs of over four billion dollars. The deleterious effects of low O_2_ in the perinatal period are thought to be the consequences of altered neural differentiation, synaptogenesis, and loss of neurons, glia and their progenitor cells due to excessive apoptosis. Recently, studies have reported significant improvement in academic functioning over time in this population, which correlated with increases in brain volume. Although encouraging, the cognitive improvement is variable and the repair/recovery mechanisms involved are undefined [1]–[11]. One possible explanation for the variable recovery from hypoxic insult in this population may be a variable response in the neurogenic zones of the brain, namely the subventricular zone (SVZ) and the subgranular zone (SGZ) [12], [13].

Currently it's appreciated that different mouse strains exhibit a wide range of phenotypes and responses to insults [12], [14]–[19]. Recently, investigators reported on mouse genomic variation and its effects on phenotypes and gene regulation, identifying the molecular foundation for understanding patterns of gene regulation [18], [19]. In this report we have observed differential responsiveness of selected mouse strains (C57BL/6 and CD1) to global hypoxic insult during the early postnatal period (P3 to P11). These differences are consistent with the poor response to and recovery from hypoxic insult noted in the C57BL/6 pups in contrast to the robust response to and recovery from hypoxic insult in the CD1 pups, mimicking the variable response/recovery observed in the human premature infant population [12], [13], [17], [20]. These findings are in contrast to previously observed strain differences noted in the C57BL/6 and CD1 adult populations following acute and chronic hypoxic insult [14], [15]. These discrepancies are likely due to differences in hypoxic protocols in addition to the significant age differences in the animals tested. Previously we noted differences in survival, apoptosis, proliferation, and differentiation of SVZ tissues, SVZ endothelial cells, and neural precursor cells that correlated with differences in GSK-3β activation and HIF-1α responsiveness to hypoxic insult [16]. Further, the differential induction and activation of HIF-1α in these strains was hypothesized to influence several downstream pathways involving NO, VEGF, BDNF and SDF-1, affecting neurogenesis and angiogenesis. These differences also correlated with behavioral differences between the two strains [12].

To better elucidate the global nature of the differences in responsiveness of these two mouse strains to hypoxic insult we harvested the SVZ tissue from P11 pups maintained under normoxic (Nx) conditions (20% O_2_) from P0 to P11 or hypoxic (Hx) conditions (10% O_2_) from P3 to P11 and utilized DNA microarray methods to identify specific genes and pathways differentially impacted by postnatal hypoxia. Our results illustrate significant differences in mRNA expression in C57BL/6 and CD1 SVZ harvested under normoxic and hypoxic conditions as well as differences between C57BL/6 and CD1 SVZ under both conditions. Differences were noted in expression of gene sets associated with Sox10-mediated neural functions, proliferation, and apoptosis between the strains. The tissue and cellular responses of these two strains to hypoxic insult may explain, in part, their differential cognitive and motor responsiveness and may promote further understanding of the variable responses of the human premature infant population.

## Materials and Methods

This study was carried out in strict accordance with the recommendations in the Guide for the Care and Use of Laboratory Animals of the National Institutes of Health. The protocol was approved by the Institutional Animal Care and Use Committee of Yale University (Protocol Number: 07366). All efforts were made to minimize suffering.

### Reagents

Antibodies directed against Sox 10 were purchased from ABCAM (www.abcam.com).

### Western Blotting

Tissues were lysed in 50 mM Tris-HCl, pH 7.4, 150 mM NaCl, 1% NP-40, 10% glycerol, 1 mM Sodium Orthovanadate, 1 mM PMSF and protease inhibitor cocktail (Roche Diagnostics GmbH, Germany). Equal amounts of total protein (10 µg) were separated by 10% or 4-20% SDS-Polyacrylamide gel, transferred to polyvinylidene difluoride membranes, and immunoblotted with antibodies according to the manufacture's instruction. Antibodies used in Western blots included antibodies directed against Sox 10 and β-actin. Bound antibodies were detected by using horseradish peroxidase-conjugated anti-IgG (Cell Signaling Technology) and a chemiluminescence detection system as previously described [12], [17], [20]. Quantitation was performed on scanned densitometric images (Epson Perfection V700 scanner with Adobe Photoshop CS, Adobe Systems, Beaverton, OR) using Quantity One software (Bio-Rad Laboratories). Western blot data are expressed as histograms of averages of relative levels (in arbitrary units) of at least three independent determinations for each protein examined.

### Mice

**Figure 1:**
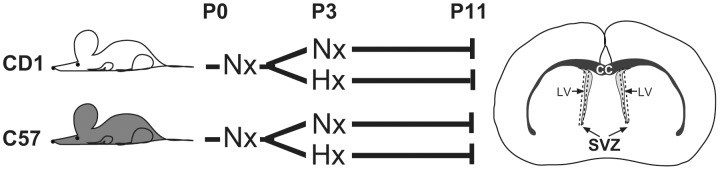
Schematic diagram depicting the experimental conditions under which the mouse pups were maintained and the areas of the brain (SVZ) isolated (dashed boxes). Nx = 20% O_2_, Hx = 10% O_2_, SVZ = subventricular zone, CC = corpus callosum, LV = Lateral Ventricle.

Timed-pregnant litters of CD1 and C57BL/6 mice were kept in normoxic conditions (20% O_2_) from birth (P0) until P3. At P3 these mice either remained in normoxic conditions or were placed in a hypoxia chamber at 10% O_2_ from P3 to P11, approximating 23 weeks gestational age to full term in the human population, as described [11], [12], [21]. At P11 the pups reared under both conditions were anesthesized, perfused with sterile PBS and their brains removed. The subventricular zones (SVZ) of three CD1 and C57BL/6 pups each from normoxic and hypoxic environments were removed (Figure 1) and mRNA was prepared [5]. RNA was extracted from SVZ tissue from 3 pups at P11 for each condition using TriZOL reagent (Invitrogen, Carlsbad, California) and further purified via Qiagen (Valencia, California) Min-elute columns. Samples were applied to the RNeasy MinElute spin columns and contaminants were eluted. High-quality RNA was eluted in water and RNA concentration was measured by using UV-Visible Spectrophotometer. 16 µg of each RNA sample was submitted to Phalanx Biotech (Palo Alto, California) for micro array analysis using the Mouse Whole Genome OneArray.

### Phalanx mouse OneArray whole Genome Microarray analysis

Each microarray contains 29,922 oligonucleotides probes (for details see www.phalanxbiotech.com). For the analysis, we restricted ourselves to 21,971 probes, which map to a unique gene locus.

### Informatics

Phalanx array data were quantile-normalized, and linear models were constructed to test for differentially expressed genes using the limma R module (http://www.bioconductor.org/packages/2.12/bioc/html/limma.html). The following contrasts (i.e. group comparisons) were tested: a) CD1 Nx vs. CD1 Hx, b) C57BL/6 Nx vs. C57BL/6 Hx, c) CD1 Nx vs. C57BL/6 Nx, and d) the differential between a) and b). For contrasts a-c, genes were selected if they exhibited an absolute fold change >2 (log2 fold change of >1) and a Benjamini-Hochberg (BH)-corrected p-value <0.01. The latter threshold was selected to control for false positive calls (see validation below). For the differential contrast d), we chose a slightly relaxed BH-corrected p-value threshold of.05 and a log2 fold change of 1.5 (absolute fold change of 2.83) for the ratio of ratios.

We performed Gene Ontology (GO)-based gene set enrichment using two programs. The R package TopGO [22] was used to derive enrichment among differentially expressed genes, using Gene-GO associations from the Phalanx array annotation file, and the “classic”/Fisher's exact test setting. In this configuration, TopGo performs a conventional enrichment test that identifies GO terms that are overrepresented among the set of significant genes. We also performed a rank-based analysis using the GSEA package [23], accessible at http://www.broad.mit.edu/gsea. Analysis was performed by downloading the java package version 2.07, and the GO and motif genes sets from the mouse Molecular Signature Database (MSigDB) version 3 (http://bioinf.wehi.edu.au/software/MSigDB/). The analysis was run using the t-statistics metric, weighted scoring scheme, and permutation of the gene sets (1000 times). GSEA evaluates the full set of ranked genes - irrespective of whether they meet a significant threshold – and determines whether there is a set of genes (for example, genes that share a particular GO term) with non-random ranks, i.e. that all have higher or lower ranks than expected by chance. In addition to GO gene sets, we used GSEA to evaluate non-random ranks of genes that are regulated by the same transcription factor (motif gene set).

In addition to TopGO and GSEA, we used the GeneMANIA web tool (http://www.genemania.org/) to generate hypotheses about regulators of the significant array genes, using GeneMANIA's built-in network mapping and function assignment algorithms [24]. For the analysis, we uploaded the list of significant genes from the differential contrast analysis (d, see above) to genemania.org, and performed an analysis using mouse co-expression networks and the generation of maximal 10 related genes. All other settings were set to default.

GeneMANIA expands the list of input genes (significant array genes) with genes that share similar functionality using information gleaned from molecular networks, such as co-expression or protein-protein interaction networks. This is particularly interesting for the discovery of regulators (for example: transcription factors) that are highly connected to the set of significant genes, particularly via gene co-expression networks. GeneMania identifies these additional genes by by “guilt-of-association”, which allows for function assignments even in absence of manual function labels. This is done by propagating gene function along networks of interaction genes, using different network types, including protein interaction networks (genes/proteins participating in the same pathway), or gene co-expression networks (reflecting genes that are regulated by the same transcription factor).

### Quantitative Real Time PCR (qRT-PCR)

In order to validate our microarray results qRT-PCR was performed on mRNA/cDNA samples isolated from the C57BL/6 and CD1. Specifically, qRT-PCR was performed on one hundred and seventy six differentially regulated following hypoxic insult in the SVZ (see Table S3). We selected genes for qRT-PCR using a two-pronged strategy: For array performance assessment, we tested all genes on top (i.e. most significantly differentially regulated) of a representative contrast, for calculation of array precision (number of validated genes divided by the number of tested genes). We thus selected the top 37 genes with significance value <0.01 in the C57 Nx vs. C57 Hx contrast (a) for qRT-PCR validation. We expanded the list of genes with qRT-PCR validation by an additional 135 genes with borderline significance but with relevant biological functions. Total RNA was isolated with TRIzol (Invitrogen, Carlsbad, CA). cDNA was prepared, starting from 1 µg of total RNA using the iScript cDNA Synthesis Kit (Bio-Rad Laboratories, Hercules, CA) according to the manufacturer's instructions. qRT-PCR was performed with SYBR Green in optimized PCR reaction conditions using the iCycler iQ system (Bio-Rad). Gapdh or B2m were used as internal controls. Data are represented as delta-Ct (ΔCt) or fold change that was calculated by the ΔΔCt method.

Isolation, culture and analysis of P0 C57BL/6 and CD1 NSC: Neural stem cells were isolated from P0 C57BL/6 and CD1 brains as previously described [12], [17], [20], [25], [26].

## Results

### Phalanx Array Analysis reveals involvement of various neural processes in C57BL/6 mice under hypoxic conditions

Differential gene expression analysis was conducted to better understand the transcriptional activity of the two mouse strains in normoxic and hypoxic conditions. Table S1 provides a complete list of the gene probesets analyzed. Specifically, we examined gene expression differences between a) C57BL/6 Nx vs. C57BL/6 Hx; b) CD1 Nx vs. CD1 Hx; c) CD1 Nx vs. C57BL/6 Nx; and d) the differential between a) and b). The corresponding ranked gene lists are illustrated in Table S2.

**Figure 2:**
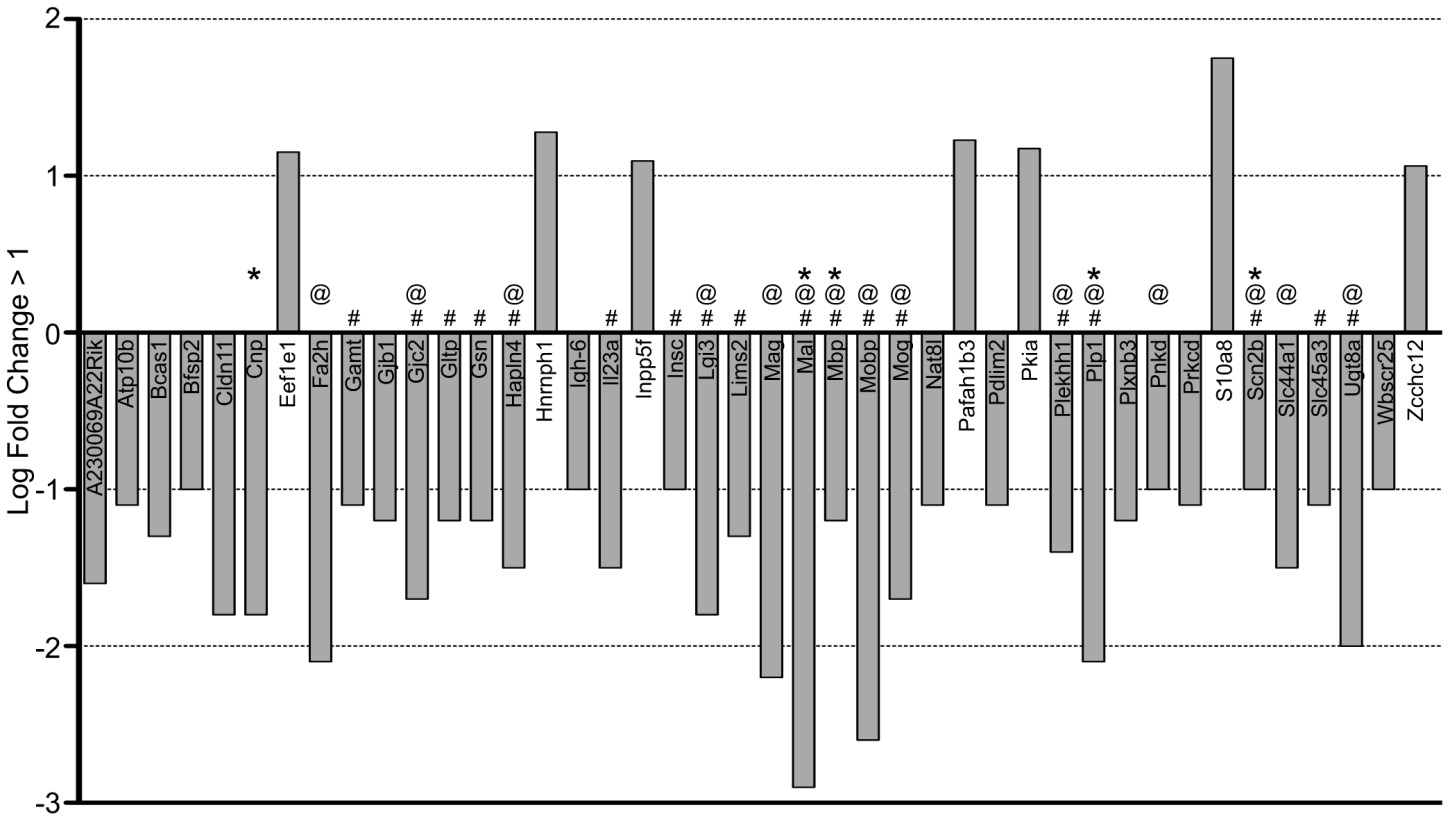
Down- and up-regulated gene probesets of SVZ tissues harvested from P11 C57 hypoxic reared (C57 Hx) compared to C57 normoxic reared (C57 Hx) pups (adjusted p-value <0.01, log fold-change >1). Analysis of these genes revealed that many were associated with aspects of neural processes and several were validated by quantitative PCR (# denotes qRT-PCR validation; @ denotes neural process related genes; * denotes GSEA Enrichment identification).

When comparing the SVZ of P11 hypoxic C57BL/6 mice to that of P11 normoxic C57BL/6 mice (a) we found 42 genes differentially regulated, 7 of which were upregulated and 35 were downregulated (Table S2, Figure 2). Eighteen of these genes were validated by qRT-PCR and/or Western blot analyses (denoted by #).

**Figure 3:**
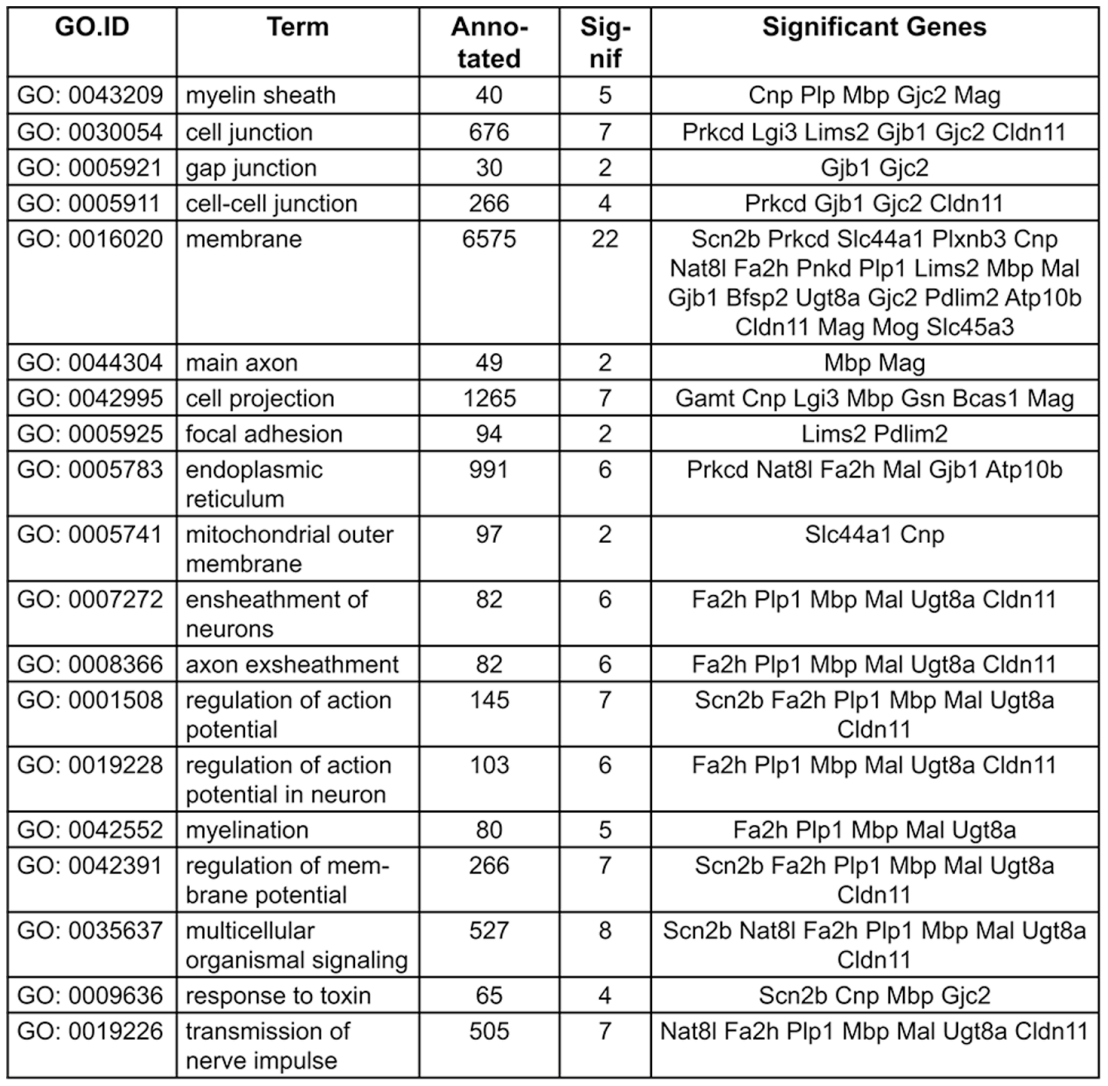
GO terms and genes common to them identified in topGO analysis illustrating down-regulated gene probesets in C57 Hx compared to C57 Nx samples ((adjusted p-value <0.01, log-fold change >1).

TopGO analysis indicates that downregulated genes in C57BL/6 Hx compared to C57BL/6 Nx samples are significantly associated (<0.05 adjusted p-value) with ensheathment of axons, regulation of action potential in neurons, regulation of membrane potential and myelin sheath including Cldn11, Gjc2, Mag, Mal, Mbp, Plp1 and Ugt8a. Other significant TopGO terms included response to toxin with Scn2b and Cnp downregulated in C57BL/6 Hx (Figure 2, denoted by @, Figure 3 & Table S2).

**Figure 4:**
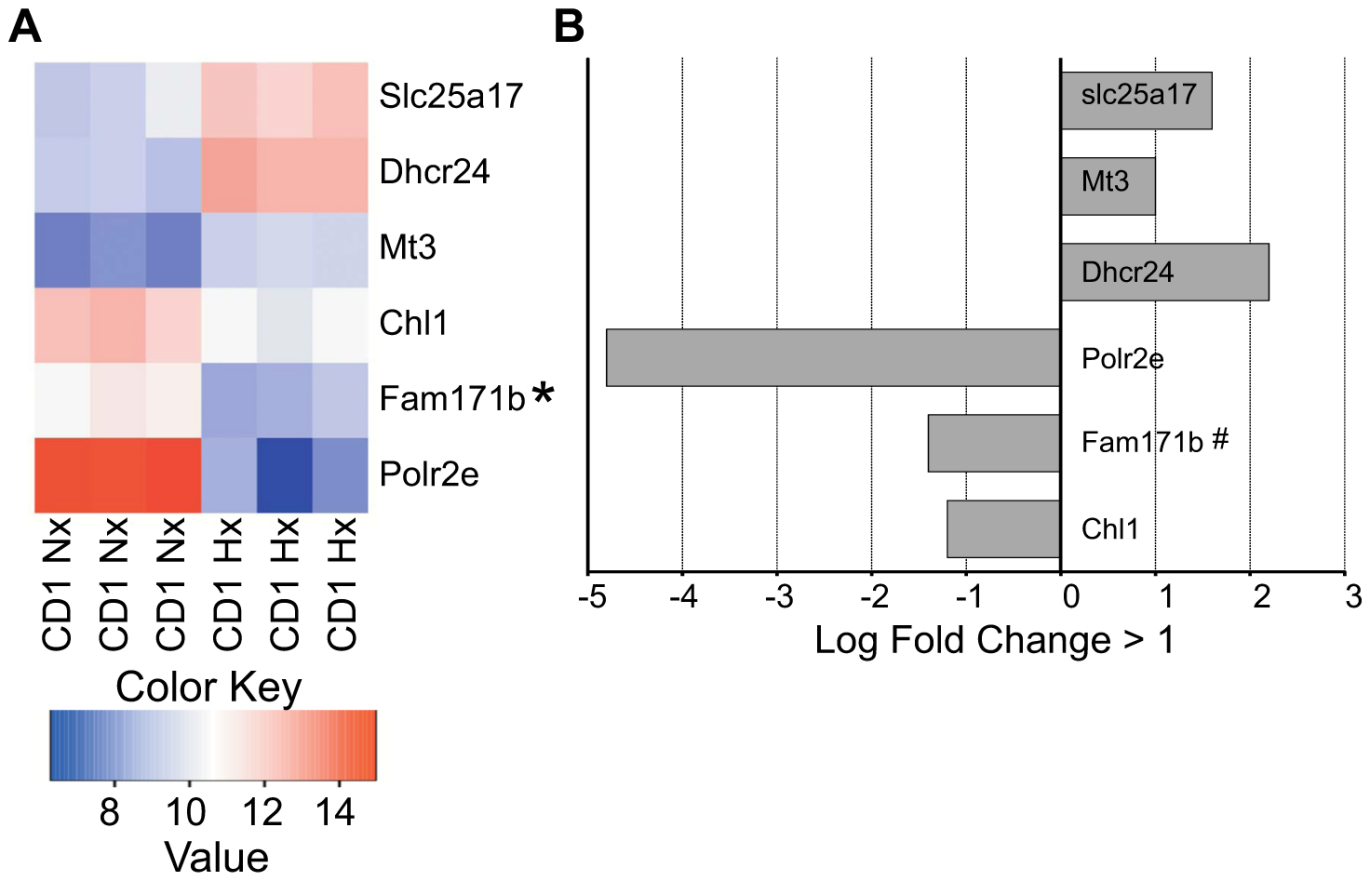
A. Heat map illustrating the triplicate, differentially expressed mRNA expression levels of SVZ tissues harvested from P11 CD1 SVZ tissues from pups held under normoxic (20% O_2_) (CD1 Nx) and hypoxic (10% O_2_) (CD1 Hx) conditions as described. Heatmap values correspond to normalized and log-transformed expression data. B. Down- and up-regulated gene probesets in the SVZ of CD1 hypoxic reared compared to CD1 normoxic reared pups (adjusted p-value <0.01, log fold-change >1). # denotes qRT-PCR validation.

In contrast we found a paucity of differentially expressed genes between CD1 mice under normoxic and hypoxic conditions (b) compared to C57BL/6 mice under the same conditions (Figure 4 & Table S2). When comparing the SVZs of P11 hypoxic CD1 mice to that of P11 normoxic CD1 mice we found six genes differentially regulated, three of which were upregulated and three were downregulated (Figure 4). Chl1, Fam171b, Dhcr24, and Mt3 are known to be involved in diverse aspects of neural processes, while Slc25a17 is involved in mitochondrial transport and Polr2e is involved in mRNA synthesis, but none of these terms attained significance in the TopGO analysis.

When comparing the normoxic states of the C57BL/6 vs. CD1 mice (c), we observed that a majority of the differentially regulated genes are expressed at higher levels in C57BL/6 mice and a minority at higher levels in CD1 mice (355 vs. 61) (Table S2). Interestingly, TopGo analysis indicated that genes with higher expression levels in C57BL/6 Nx compared to CD1 Nx samples are significantly (<0.05 adjusted p-value) associated with the myelin sheath: Gjc2, Mag, Mbp, PLP1, Serinc5, and Tspan2; and with regulation of action potential (<0.2 adjusted p-value), including Plp1, Mbp, Mal and Cldn11.

TopGO did not find any significant GO terms linked to genes (<.2 adjusted p-value) with higher expression levels in CD1 Nx compared to C57BL/6 Nx samples. The top terms were linked to negative regulation of protein serine/threonine kinase activity, neural crest cell differentiation, and negative regulation of leukocyte proliferation, including Lrp6, Sfrp1, and Sox 11 and Gstp1 (Table S2).

**Figure 5:**
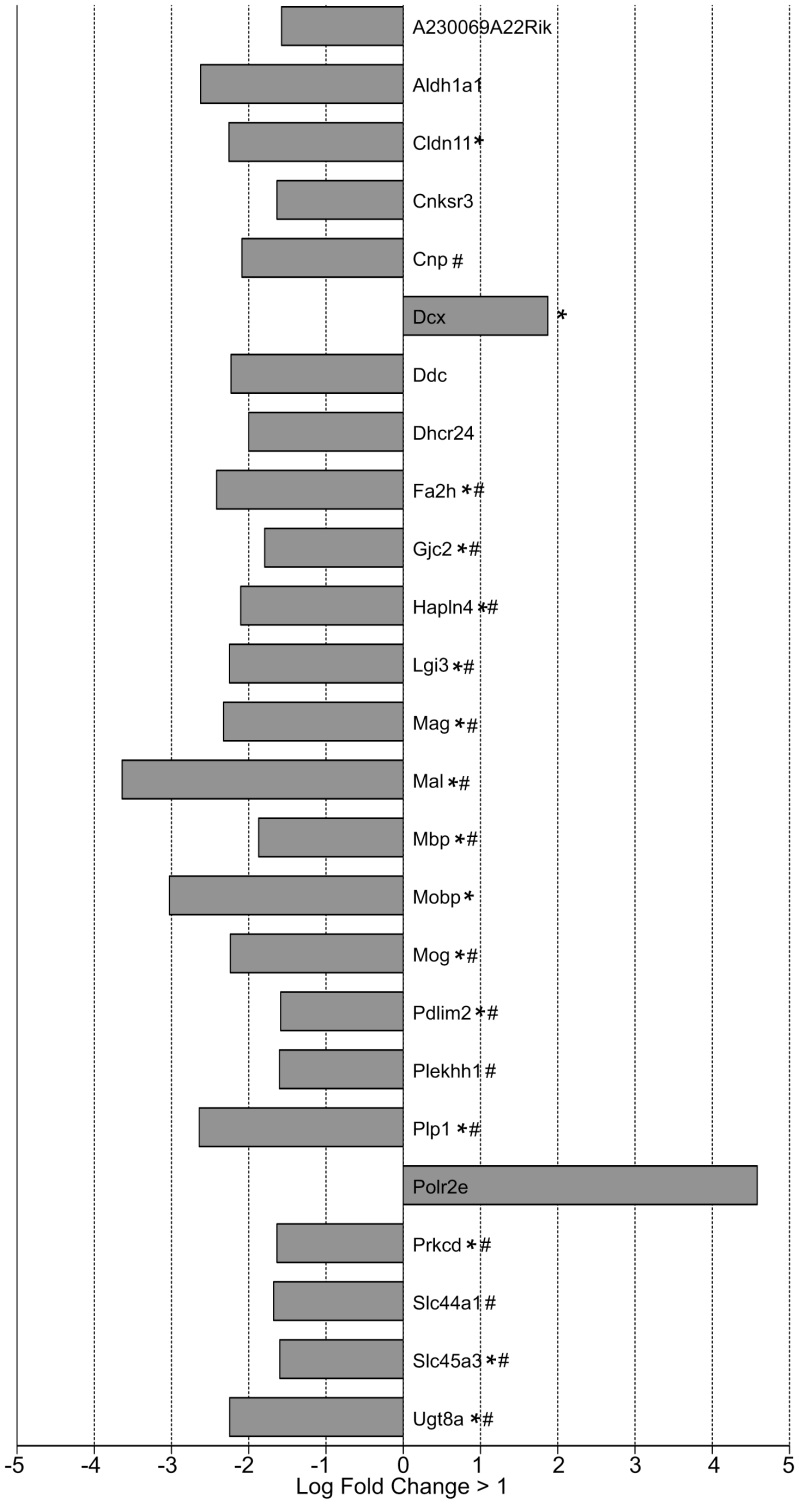
Statistically significant gene probesets from differential contrast: differentially regulated genes in SVZ of [C57 normoxic reared compared to C57 hypoxic reared P11 pups] *versus* [CD1 normoxic reared compared to CD1 hypoxic reared P11 pups]. (Adjusted p-value <0.05, log fold-change >1.5 or <−1.5). (# denotes qRT-PCR validation; Asterisks denote neural process related genes).

**Figure 6:**
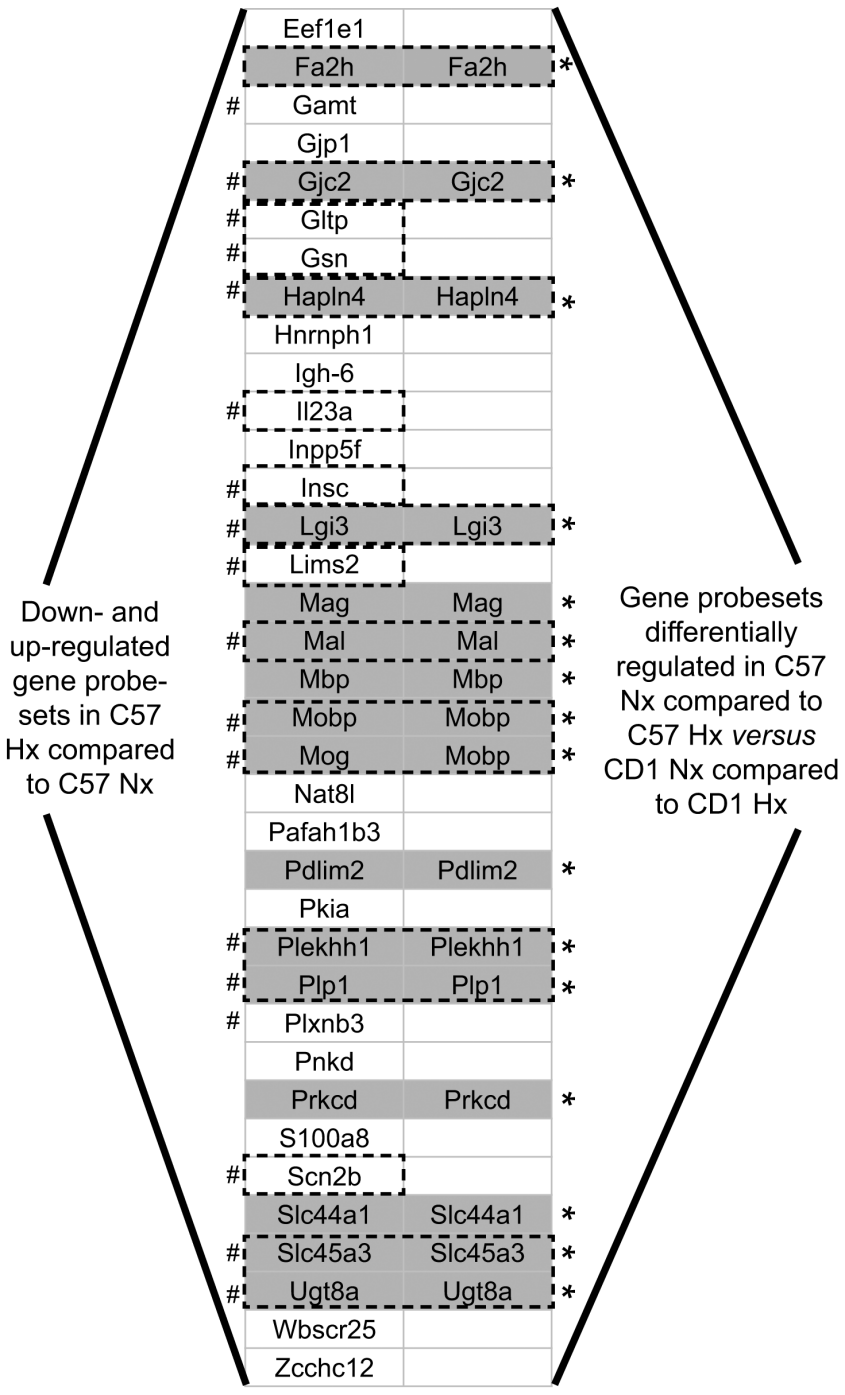
Summary of the genes associated with aspects of neural processes (*) including genes associated with: ensheathment of axons, regulation of action potential in neurons, myelin sheath, response to toxin, myelination, structural molecule activity, regulation of membrane potential, wide pore channel activity, and responses to toxin. These genes were validated by quantitative PCR and were found to be common in C57 Nx vs. C57 Hx (left column) and found to be differentially regulated in the differential contrast samples (right column). (# denotes qRT-PCR validation; Asterisks denote neural process related genes).

The differential contrast (d) is dominated by genes that are downregulated in C57BL/6 mice (Figure 5). (Table S2, Figure 5). TopGO analysis (Table S2) revealed that many of the significant GO terms (p-value <0.05) were associated with genes involved in neural processes such as ensheathment of axons, regulation of action potential in neurons, myelin sheath, response to toxin, regulation of membrane potential (Figure 2 & Figure 5, marked by asterisks). Several of these genes were validated by quantitative PCR (Figure 5, marked by the # symbol) as summarized in Figure 6. Genes that show significance in the differential contrast include many genes that are downregulated in C57BL/6 Hx compared to C57BL/6 Nx, and show no significant expression changes in CD1, including Mag, Mal, Mobp, Gjc2, and Ugt8a. Other genes such as DCX, a known marker of neurogenesis, show opposite regulation between C57BL/6 and CD1 in the differential contrast (Figure 5, Table S2).

The data suggests that these downregulated genes may be responsible for our previously observed phenotypic differences between these strains [12], [13], [16], [17], [20].

### Array Validation

The quality of the array data was assessed by selectively examining 37 of the differentially expressed genes (adjusted p-value <0.01) in C57BL/6 Nx vs. C57BL/6 Hx using qRT-PCR. Twenty-three of those genes (62.1% precision) were validated. We also performed qRT-PCR on an additional 135 genes of interest that showed borderline significant differential expression. Of those, 29 were validated (Table S3).

**Figure 7:**
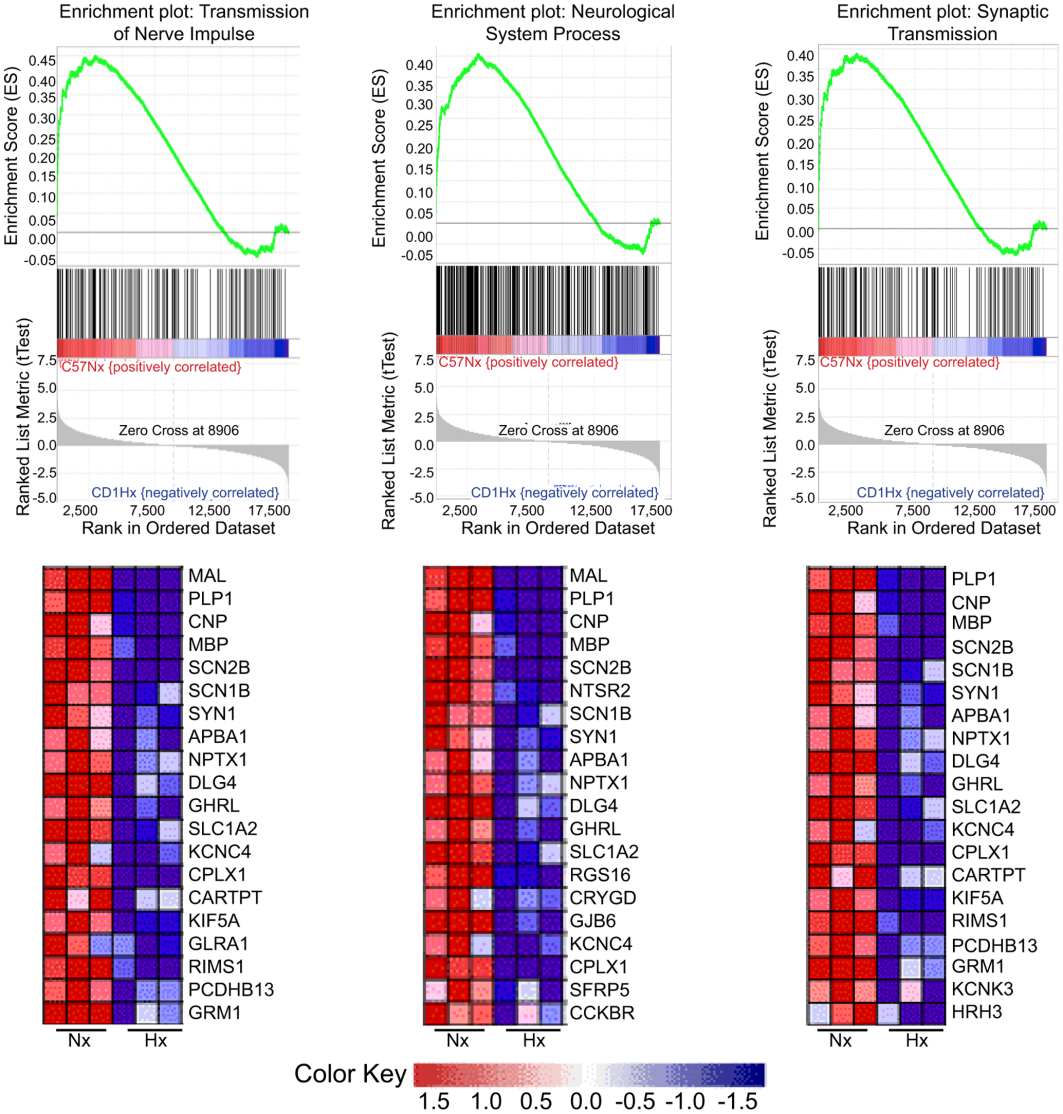
Leading edge genesets (Transmission of Nerve Impulse, Neurological System Process, System Process and Synaptic Transmission) for C57 Nx vs. C57 Hx identified by GSEA analysis. This figure illustrates genes common to these genesets that were decreased following hypoxia in C57 SVZ. The genes, which are associated with neural processes, common to these gene subsets include MAL, PLP1, CNP, MBP, SCN2B, SCN1B, SYN1, APBA1, DLG4, GHRL, SLC1A2, CPLX1 and CARTPT.

Complementary analysis utilizing Gene Set Enrichment analysis (GSEA): Enrichment analysis was undertaken to analyze differences between the hypoxic responses of these two mouse strains. The results reinforce the involvement of processes involving the neural processes for C57BL/6 mice under hypoxic conditions (Figure 7). Similar to TopGO, the GSEA analysis revealed down-regulated gene probesets in C57 Hx compared to C57 Nx samples again highlighting the role of neural processes. 577/975 GSEA GO gene sets analyzed were downregulated in hypoxic SVZ tissue of C57BL/6 pups. Of those 577 gene sets, 65 were significant at FDR <25%. Of these we selected three of the 30 leading edge gene sets: transmission of nerve impulse (third), synaptic transmission (eighth) and neurological system process (tenth) to analyze further in light of the persistent cognitive deficits observed in C57BL/6 mice reared in hypoxia from P3 to P11 [12]. Several down-regulated genes were found to be common among the gene sets selected (Figure 7). PLP1 and MBP were noted to be down regulated and common to all three gene sets analyzed as were while CNP, SCN2B, SCN1B, APBA1, NPTX1, DLG4, GHRL, SLC1A2, KCNC4, CPLX1 and CAARTP. CARTPT, KIF5A, RIMA1, PCDHB13 and GRM1were down-regulated in both the transmission of nerve impulse and synaptic transmission genesets, while SYN1 was down regulated in both the transmission of nerve impulse and neruological system process genesets. KCNK3 and HRH3 were down regulated in the synaptic transmission geneset. The member genes common to these gene sets include genes implicated in myelin biogenesis and/or function (MAL); a major constituent of the myelin sheath (MBP); a myelin-associated enzyme that makes up 4% of total CNS myelin protein (CNP); voltage-gated sodium channels (NaV) that are responsible for action potential initiation and propagation (SCN1b & 2b); members of the synapsin gene family (SYN1); a member of the X11 protein family (APBA1), which is a neuronal adapter protein; a member of the neuronal pentraxin gene family (NPTX1), which may play an important role in synaptic remodeling; a member of the membrane-associated guanylate kinase (MAGUK) family (DLG4), whose overexpression or depletion changes the ratio of excitatory to inhibitory synapses in hippocampal neurons; Ghrelin (GHRL), a gene encoding ghrelin-obestatin preproprotein, which generates ghrelin and obestatin, which are thought to be involved in inhibiting thirst and anxiety, improving memory, and regulating sleep; CPLX1, a cytosolic complexin encoded by the complexin/synaphin gene family that functions in synaptic vesicle exocytosis; and CARTPT, a cocaine- and amphetamine-regulated transcript protein thought to promote neuronal development and survival in vitro. Of these genes Carpt, Cnp, Mal, and SCN2b were validated by qRT-PCR.

Conversely, the 503 GSEA gene sets down-regulated in CD1 pup hypoxic SVZ tissue, did not include genes associated with transmission of nerve impulse, regulation of action potential, neurological system processes, synaptic transmission and system processes at a significant threshold of FDR <25% (data not shown). Thus, the differences in the reductions in genes comprising neuronal and myelin components in the C57BL/6 pups correlates with the persistent behavioral deficits previously noted in this strain compared to the CD1 mice [12]. (Table S3).

### Gene co-expression analysis reveals the involvement of the SOX10 transcription factor in regulating gene expression in C57BL/6 mice under hypoxia

**Figure 8:**
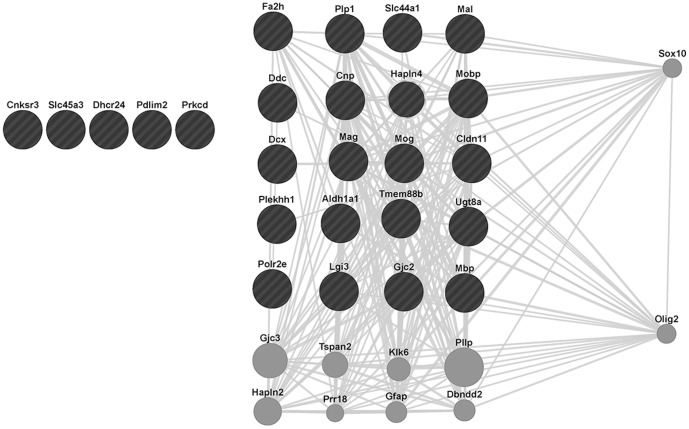
GeneMANIA-generated network of the queried genes (down-regulated gene probesets in C57 Hx compared to C57 Nx, listed in Table S2) illustrating the relationships of these genes with Sox10. The black circles denote the subset of queried genes that are known to exhibit co-expression with Sox10 (gray lines),. The grey circles denote other genes that are known to exhibit these relationships with Sox10 including Olig2, which also exhibits co-expression with several of the genes in our query set. Genes in our query set which did not exhibit co-expression with Sox10 or Olig2 are represented without any connecting gray lines.

The differential contrast highlights a distinct set of genes that are being downregulated in C57BL/6 mice (Table S2). In order to investigate the possibility of the involvement of a particular transcription factor (TF) in the regulation of these genes, we conducted both a GSEA TF target gene set enrichment and a GeneMania-based analysis using co-expression network-based gene set expansion. The GSEA analysis (using the motif gene sets) did not reveal any significant TF target gene set (data not shown) for these differentially regulated genes. In contrast, using GeneMANIA's function propagation along gene co-expression networks identified the TFs Sox10 and Olig2 as functionally related to genes that are identified in the differential contrast (Figure 8). As function propagation was conducted via gene co-expression networks, the question arises whether these TFs are direct regulators of these genes. Indeed, review of ChIP experiments and existing TF target databases [27]–[29] for Sox10 indicate that approximately one third of the differentially expressed genes (predominantly downregulated in C57BL/6 Hx) have been experimentally found to be regulated by SOX10, namely PLP1, MAG, GJC2, MBP, UGT8, CNP and GJB1. The negative GSEA result is explained by the lack of a curated SOX10 target gene set. Further analysis revealed that one of the genes that appears to be regulated by Sox10, Olig2 [30], [31], is also a potential regulator of several of our query listed genes. This too is consistent with Sox10's role as a direct and indirect regulator of the differential hypoxic responses observed in these two mouse strains.

**Figure 9:**
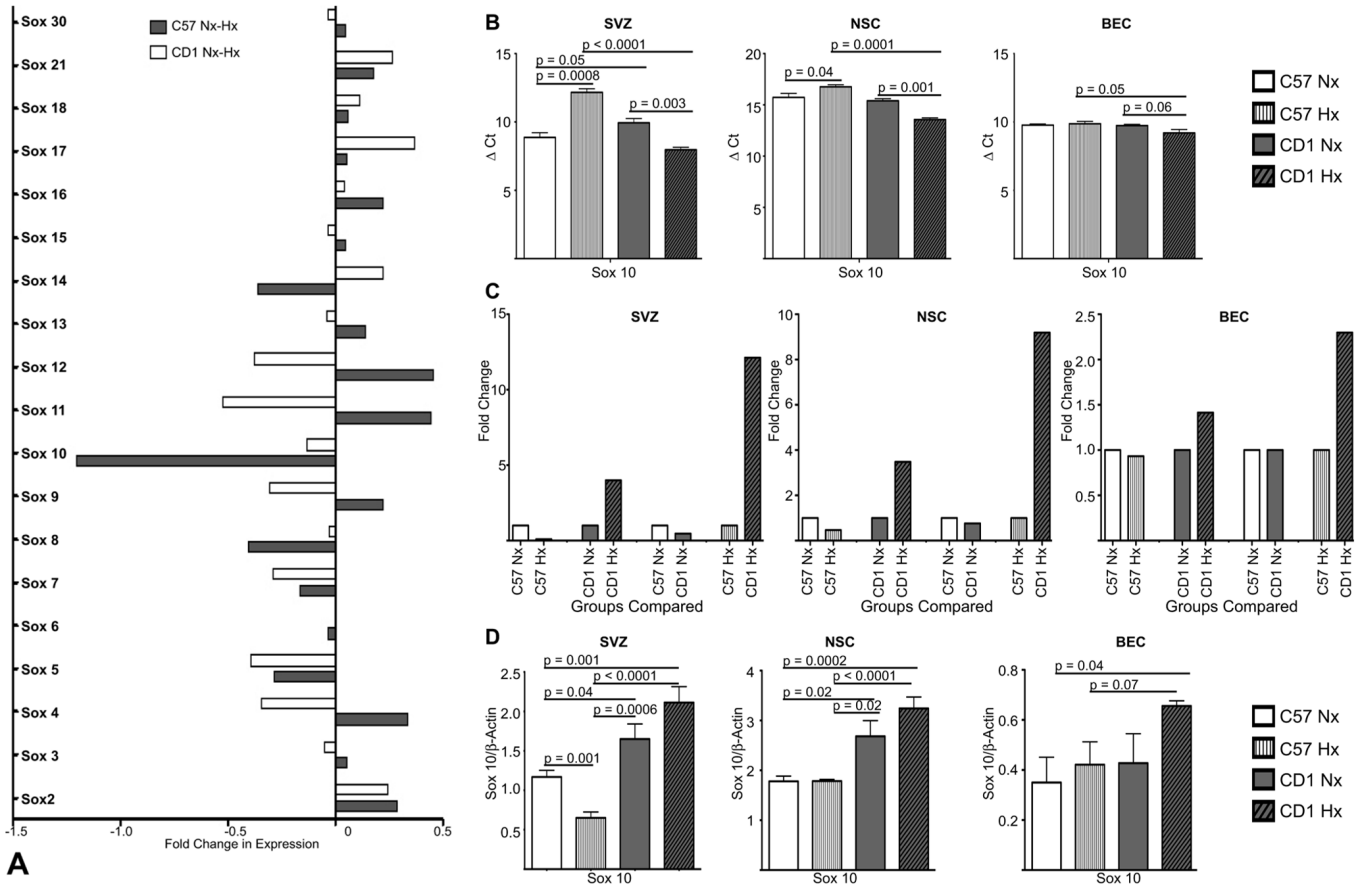
Differential expression of Sox family members and Sox10 in C57 and CD1 under normoxic and hypoxic conditions. A. Down- and up-regulated Sox gene family probesets in C57 Hx compared to C57 Nx (white bars) and CD1 Hx compared to CD1 Nx (shaded bars) (adjusted p-value <0.01, log fold-change >1) SVZ. Of the Sox family member genes analyzed only Sox10 mRNA was significantly decreased in C57 SVZ samples. B. Transcript levels of Sox10 were evaluated by qRT-PCR in normoxic and hypoxic treated C57 and CD1 pups (SVZ), and cultured NSC and BEC. In response to Hx, Sox10 mRNA expression decreased in C57 SVZ and NSC and increased CD1 SVZ and NSC, n = 3. C. A ΔΔCt method was used to determine fold changes between C57 and CD1 normoxic and hypoxic treated SVZ, NSC and BEC Sox10 transcript levels. This analysis illustrated decreased Sox10 mRNA expression in Hx C57 SVZ and NSC and increased expression in CD1 SVZ and NSC. n = 3. D. Western blot analysis of Sox10 protein levels in normoxic and hypoxic treated C57 and CD1 pups (SVZ), and cultured NSC and BEC revealed decreased Sox10 protein in Hx C57 SVZ and increased Sox10 protein in Hx CD1 SVZ, NSC and BEC. n = 3.

Examination of our array analysis revealed that of the Sox family members assessed in the array, only Sox10 was significantly (with p-value <0.05) down-regulated in C57 Hx SVZ compared to C57 Nx SVZ with no down-regulation noted in the CD1 SVZ (Figure 9a). qRT-PCR and Western blot analysis of Sox 10 mRNA levels and protein expression confirmed the array data findings (Figure 9b-d). Specifically, ΔCt and fold change analysis revealed a decrease in Sox 10 mRNA in response to hypoxia in the C57BL/6 tissue while Sox 10 mRNA increased following hypoxia in the CD1. Similar results were observed in Sox 10 protein expression. Considering Sox10's known functions in the survival, maintenance of pluripotency and differentiation of neural precursors, specifically directing neural stem cells toward the oligodendrocyte lineage [32], this down-regulation in C57 Hx SVZ is consistent with the C57 SVZ's poor response to and recovery from hypoxic insults [12], [13], [16], [17]

## Discussion

The wide range of responsiveness to and recovery from hypoxic insult in the human premature newborn population prompted us to develop murine models to study this process. Both our in vivo and in vitro models have shown mouse strain differences in responsiveness to and recovery from a hypoxic insult that mimics the human experience [12], [16]. Our previous studies were based on cell biological and animal studies and literature that has indicated particular targets likely to be involved in the hypoxic response [12], [16], [17], [20]. In this report, using an unbiased microarray-based approach, we discovered specific differences in a number of genes involved in Sox10-mediated neural function and the regulation of survival, and proliferation. Further, the strain specific differences in gene expression observed are consistent with our reported neurogenic, behavioral, and biochemical differences noted in these two strains [32], [33]. Specifically, the strain exhibiting acute and chronic behavioral problems (as evidenced by hyperactivity acutely and changes in spatial memory, callosal connectivity, and behavioral lateralization of the hemispheres) (C57BL/6) [12], [17] was the one that exhibited decreases in expression of genes common to these selected gene sets. Further, the strain exhibiting only mild, transient behavioral problems (CD1) [12], [17] exhibited much fewer changes in gene expression.

Importantly, we have identified differences in regulating gene sets subserving various Sox10-mediated neural processes [34], including axonal ensheathment, nerve impulse transmission, regulation of action potential, and synaptic transmission. The hypoxia-induced down-regulation of these genes in the C57BL/6 relative to CD1 is congruous with the impaired neurogenesis/oligodendrocytogenesis and defective behavior observed in this strain. Additionally, a number of genes associated with apoptosis and proliferation also exhibited altered expression in the CD1 strain, cnsistent with maintenance of higher proliferative and lower apoptotic rates in a hypoxic environment. Interestingly, we have found significant differences in expression of Sox10, a known to regulator of various aspects of oligodendrocyte precursor differentiation and oligodendrocyte development [34]–[36]. Sox10 appears to be a key modulator of a number of the differentially expressed genes identified in this study. Further, Sox10 mRNA and protein decreases in response to hypoxia in the C57BL/6 SVZ, while hypoxia triggers an increase in Sox10 mRNA and protein in the CD1 SVZ. Because Sox10 is a key regulator of numerous genes important for the response to and recovery from hypoxia, and is reduced in the C57BL/6 mice, this suggests Sox10 may be a key contributor to the differences observed between these strains affecting oligodendrocyte precursor differentiation and oligodendrocyte development [34]–[36].

An interesting finding was the lack of correlation of some of our previous biochemical data with the differential gene expression in the array analyses. Namely, increases in selected proteins including HIF-1α, PHD2, VEGF, BDNF, β-catenin and GSK-3β were not identified in this array. However this apparent discrepancy is likely explained by differences in mRNA and protein expression kinetics, half-life and degradation [14], [37]. For example, we have documented differential ratios of active and inactive GSK-3β in the SVZ and SVZ-derived cells as well as different ratios of cytoplasmic- and nuclear-localized HIF-1 and 2α and PHD2 in cultured NSC from these two strains without appreciable changes in mRNA levels [12], [16].

In aggregate, these data support the concept that strain differences are important determinants when modeling diseases in mice and may be useful as tools in understanding the variability of responsiveness to and recovery from a particular insult. The unbiased array studies presented in this report illustrate previously unrecognized murine strain differences in several Sox10-mediated neural gene sets following hypoxic insult. When considered with the previously confirmed strain differences in response to and recovery from hypoxia, these findings may shed light on mechanisms underlying this differential outcome between strains.

Further interrogation of the differentially expressed gene sets will provide a more complete understanding of the differential responses to and recovery from hypoxic insults in selected murine strains. This will allow for more informed modeling of the ranges of disease severity observed in the very premature human population.

## Supporting Information

Table S1 Complete list of 21,971 genes (probesets). Output format corresponds to the limma write.fit command, showing the average log-intensity (A), the log-ratios/fold-change (Coeff), moderated t-statistics (t), t-statistic P-values (p-value), Benjamini-Hochberg adjusted p-values (p.value.adj) for the following contrasts: C57NX vs. C57HX, CD1NX vs. CD1HX, C57NX vs. CD1 NX, and differential contrast (C57NX vs. C57HX) vs. (CD1NX vs. CD1HX).
(XLSX)

Table S2 *Panel A* shows Differentially expressed genes (probesets) for: C57NX vs. C57HX, CD1NX vs. CD1HX, C57NX vs. CD1 NX, and differential contrast (C57NX vs. C57HX) vs. (CD1NX vs. CD1HX). Output format corresponds to the limma write.fit command, showing the average log-intensity (A), the log-ratios/fold-change (Coeff), moderated t-statistics (t), t-statistic P-values (p-value), Benjamini-Hochberg adjusted p-values (p.value.adj), and gene annotation. *Panel B* shows significantly enriched topGO categories for the genes with differential expression.
(XLSX)

Table S3 *Panel A* shows RT-PCR validation of genes with differential array expression. *Panel B* shows additional validated candidate genes.
(XLSX)
